# Strategies That Facilitate Extraction-Free SARS-CoV-2 Nucleic Acid Amplification Tests

**DOI:** 10.3390/v14061311

**Published:** 2022-06-15

**Authors:** David J. Delgado-Diaz, Dhanasekaran Sakthivel, Hanh H. T. Nguyen, Khashayar Farrokzhad, William Hopper, Charles A. Narh, Jack S. Richards

**Affiliations:** 1 ZIP Diagnostics Pty Ltd., Collingwood, VIC 3066, Australia; dhana.s@zipdiag.com (D.S.); hanh.n@zipdiag.com (H.H.T.N.); khashayar.f@zipdiag.com (K.F.); bill.h@zipdiag.com (W.H.); charles.n@zipdiag.com (C.A.N.); jack.r@zipdiag.com (J.S.R.); 2 Department of Medicine, University of Melbourne, Melbourne, VIC 3010, Australia; 3 Department of Infectious Diseases, Monash University, Melbourne, VIC 3004, Australia

**Keywords:** COVID-19, SARS-CoV-2, nucleic acid extraction, diagnostics, NAAT, isothermal amplification, RT-PCR

## Abstract

The COVID-19 pandemic has resulted in an unprecedented global demand for in vitro diagnostic reagents. Supply shortages and hoarding have impacted testing capacity which has led to inefficient COVID-19 case identification and transmission control, predominantly in developing countries. Traditionally, RNA extraction is a prerequisite for conducting SARS-CoV-2 nucleic acid amplification tests (NAAT); however, simplified methods of sample processing have been successful at bypassing typical nucleic acid extraction steps, enabling extraction-free SARS-CoV-2 NAAT workflows. These methods involve chemical and physical approaches that are inexpensive and easily accessible alternatives to overcome extraction kit supply shortages, while offering acceptable test performance. Here we provide an overview of three main sample preparation strategies that have been shown to facilitate extraction-free SARS-CoV-2 NAATs.

## 1. Introduction

Severe acute respiratory syndrome coronavirus 2 (SARS-CoV-2) is a positive sense, single-stranded RNA virus with a genome size of 30 kb, encoding 16 non-structural proteins associated with viral replication, four structural proteins (spike, envelope, membrane, and nucleocapsid) and eight accessory proteins [1,2,3]. A number of these proteins and the genes that encode them have been utilised as diagnostic markers for SARS-CoV-2 infection. The majority of diagnostic tests for SARS-CoV-2 fall under three categories—nucleic acid-based tests (NAATs), antigen, and antibody detection tests. Detection of the viral RNA in respiratory samples using real-time reverse transcriptase—polymerase chain reaction (RT-PCR), is regarded as the gold standard for COVID-19 diagnosis [4]. However, widespread deployment of RT-PCR testing worldwide has caused a backlog of testing and supply chain disruption, impacting public health responses [5]. A critical bottleneck for RT-PCR testing is sample processing and RNA isolation, which are laborious and expensive. Therefore, the development and validation of extraction-free SARS-CoV-2 NAAT protocols can potentially facilitate a more rapid deployment of simple and supply chain resilient SARS-CoV-2 NAATs [6].

## 2. Extraction-Free Strategies for the Detection of SARS-CoV-2 by NAATs

The majority of SARS-CoV-2 NAAT assays, including RT-PCR, loop-mediated isothermal amplification (LAMP), and other isothermal platforms are lab-based and require the extraction of viral RNA from crude specimens using commercially available nucleic acid extraction kits prior to amplification. These kits purify RNA from all the other components of the sample including inhibitors, that may otherwise interfere with the amplification of gene targets. In well-resourced laboratories, a large proportion of the testing workflow including sample processing, RNA extraction, and amplification has been automated on robotic platforms to increase testing throughput. However, the use of these robotic platforms is limited and unfeasible in low- and middle-income countries due to their high costs.

**Figure 1:**
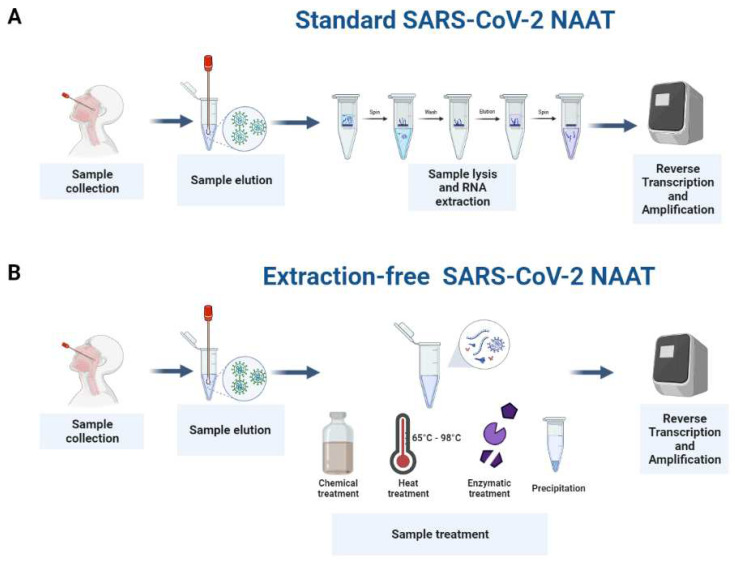
Standard vs. extraction-free SARS-CoV-2 NAATs. Standard SARS-CoV-2 NAAT workflows involve the collection, elution, and lysis of respiratory samples, and extraction of their RNA prior to reverse transcription and amplification of the target (**A**,**B**). Alternative workflows that bypass the extraction step and streamline the procedure involve chemical and physical treatment methods that inactivate inhibitory or interfering substances but do not impact the integrity of the nucleic acid or the amplification of the target.

Instead, alternative extraction-free SARS-CoV-2 NAAT assays that only require simpler workflows have the potential to streamline and reduce the cost of COVID-19 diagnosis (Figure 1). In order to match the advantages that RNA extraction provides, there are three main issues that extraction-free SARS-CoV-2 NAATs must overcome: (1) to enrich the target template into a smaller final working volume; (2) to remove the contaminants which may interfere with the amplification; and (3) to prevent the degradation of the target template by removing DNases and RNases.

### 2.1. Enrichment of Template

#### 2.1.1. Adjusting the Proportion of Sample in the Reaction Mixture

The proportion of samples in the reaction mixture can be increased by simply adding more unprocessed samples to the NAAT assay. For instance, Kriegova, Brown, and colleagues reported successful detection of SARS-CoV-2 RNA in unprocessed nasopharyngeal, nasal, and oral swabs by increasing the sample input from 8–10% to 40–47% of the total RT-PCR reaction mixture [7,8]. The rationale behind this strategy is based on the notion that increased template input accelerates reaction kinetics. However, the presence of contaminants and inhibitors in the non-processed samples can inhibit or delay the NAATs impacting the assay sensitivity and specificity [9]. As a result, it is unlikely that this method will be successful as a standalone method for extraction-free SARS-CoV-2 NAAT.

#### 2.1.2. Precipitation and Concentration of Nucleic Acid

Nucleic acid precipitation concentrates the target template and removes inhibitors without requiring commercial kit reagents or spin columns. SARS-CoV-2 RNA precipitation using polyethylene glycol has been shown to successfully enrich the viral template for RT-PCR to the same standard as samples extracted by the automated NucliSENS^®^ easyMAG^®^ platform (bioMérieux, Boxtel, The Netherlands) [10]. Similarly, isopropanol precipitation of SARS-CoV-2 nucleic acid present in heat-inactivated nasopharyngeal swab eluates has provided sensitivities comparable to a standard RT-PCR workflow [11]. While nucleic acid precipitation may provide a cost-benefit, it remains a lengthy and complex procedure which requires operators with a high level of expertise, dedicated laboratory space and equipment such as high-speed centrifuges.

### 2.2. Dilution and Removal of Contaminants That May Interfere with NAAT

Respiratory specimens, routinely used for COVID-19 identification, contain water, ions, mucins and other bioactive macromolecules [12] as well as known PCR inhibitory chemicals present in transport buffers [13]. Some of these compounds can inhibit or interfere with NAATs if left unprocessed or untreated. Grant, Wee, Lee, Morecchiato, and colleagues overcame the issues associated with using crude samples by either reducing the input volume to 4–5% of the total reaction mixture or by diluting the sample by a factor of 4–5 prior to its addition to the NAAT master mix [14,15,16,17].

Additionally, the use of chemicals such as Chelex-100 (Bio-Rad, Hercules, CA, USA), a chelating resin used to assist in the removal of extra metal ions, has been successfully used in combination with other methods of sample preparation (such as addition of RNAse inhibitors or heat treatment) for the detection of SARS-CoV-2 RNA in saliva and nasopharyngeal swabs using several NAAT platforms including RT-PCR, RT-ddPCR, and RT-LAMP [17,18,19,20]. The use of enzymes with improved tolerability to inhibitors is another approach that may facilitate the use of crude respiratory specimens to detect SARS-CoV-2 RNA in the absence of RNA extraction [7].

### 2.3. Prevention of Template Degradation

#### 2.3.1. Chemical Treatment of Respiratory Samples

Treatment of respiratory specimens with ribonuclease (RNase) inhibitors to minimize the impact of RNases that degrade the viral RNA has also been reported, although this strategy is often combined with other approaches [15,21]. For instance, the use of the RNAse inhibitor RNAseOUT™ (Thermo Fisher Scientific, Waltham, MA, USA) has been combined with dilution of the sample [15] and with the addition of carrier-RNA [22].

Reducing agents such as tris (2-carboxyethyl)phosphine (TCEP) and Dithiothreitol (DTT), present in products such as RNasecure (Thermo Fisher Scientific, Waltham, MA, USA) and Mucolyse (Pro-Lab Diagnostics, ON, Canada) have also been useful to process respiratory specimens and facilitate NAAT by solubilizing mucus strands and by reducing inhibitors of protein origin [19,20,23]. Rabe et al. found that a combination of TCEP/EDTA (2.5 mM/1 mM) reduced the mucus viscosity of the specimens, denatured proteins, and facilitated the detection of SARS-CoV-2 RNA by RT-LAMP [23]. Similar to TCEP and DTT, Sputasol (Oxoid, Basingstoke, England) has facilitated the processing of sputum and nasal exudates for SARS-CoV-2 RT-PCR applications [15].

#### 2.3.2. Enzymatic Treatment of Respiratory Samples

Pre-treatment of samples with proteolytic agents such as proteinase K emerged as another strategy to inactivate the inhibitory proteins and enzymes present in respiratory samples, and to simplify SARS-CoV-2 NAATs [21,24,25,26,27,28]. Proteinase K treatment of nasopharyngeal and oropharyngeal swabs samples allowed successful detection of SARS-CoV-2 RNA in nasopharyngeal and oropharyngeal swabs treated with proteinase K [24]. However, a major setback of this strategy is the incubation of the sample at 37–56 °C and the requirement of a heat inactivation step prior to sample addition, to prevent degradation of reverse transcriptase and DNA polymerases.

#### 2.3.3. Heat Treatment of Respiratory Samples

In addition to being used as a biosafety measure, to inactivate potentially infectious samples, heat treatment has been reported to be compatible and simplify both SARS-CoV-2 RT-PCR and isothermal amplification [13,29,30]. Even though this sample treatment approach has been reported several times, there is no consensus on the ideal heat treatment conditions necessary to process respiratory samples for efficient amplification of SARS-CoV-2 RNA. Reported heat treatment conditions include temperatures that range from 65 to 98 °C and treatment times of 5 to 30 min [10,13,29,30,31,32,33,34]. However, this temperature range is inconsistent with Burton and colleagues’ findings, who reported a reduction in SARS-CoV-2 PCR sensitivity after heat treating specimens at temperatures higher than 80 °C but not at temperatures lower than 60 °C [35]. Therefore, it is important to consider that a consistent and efficient strategy based on heat treatment might require fine-tuning as heating devices and their heat exchanging properties, as well as volume of sample processed, differ in all these reports and could be a determinant of the success of the assay.

Similar to heat treatment, thermal shock treatment of the samples prior to SARS CoV-2 NAAT has been reported by Blairon and colleagues. This workflow involves subjecting nasopharyngeal swab eluates to a thermal shock consisting of 95 °C for 5 min followed by active cooling at 4 °C for 10 min [36].

A combination of the above-mentioned methods of sample processing have also been successful in providing detectable template [10,13,15,21,22,23,30,31,37]. However, a comparative study to evaluate the diagnostic performance of these methods is essential, as these have not been studied in parallel.

#### 2.3.4. Optimization of Amplification Conditions

Finally, another interesting approach used to optimize SARS-CoV-2 NAATs of unextracted samples relies on modifying PCR cycling conditions such as the annealing and extension times [38]. In this regard, Lownik and colleagues found that a 10-second annealing/elongation time per PCR cycle resulted in a shorter time to result for unextracted nasopharyngeal specimens [38].

## 3. Discussion

SARS-CoV-2 RT-PCR remains the gold standard test for the diagnosis of SARS-CoV-2 infections; however, other NAAT platforms that require less sophisticated instruments but provide similar performance to RT-PCR such as LAMP, Recombinase Polymerase Amplification (RPA), and Nicking and Extension Amplification Reaction (NEAR) are available. Shortages and the high cost of SARS-CoV-2 NAAT kits and associated supplies have affected their deployment and distribution worldwide, hampering transmission control efforts. Streamlining SARS-CoV-2 NAAT workflows by simplifying the extraction of RNA from respiratory specimens have the potential to make NAATs faster, more accessible and deployable, and less sensitive to supply chain shortages.

Several sample processing methods that bypass the typical RNA extraction step have been reviewed here (Table 1). These physical and chemical methods of sample preparation offer alternatives to the expensive and often limited RNA extraction kits, which have been in shortage during the early stage of the COVID-19 pandemic. They also offer a simpler sample preparation workflow with fewer handling steps which can be aimed at more diverse testing settings, especially in low- and middle-income countries.

These extraction-free sample preparation strategies are not without their limitations. In most cases, several physical and chemical methods need to be used together to tackle the multi-faceted challenges of neutralising inhibitors while still being able to achieve acceptable assay sensitivity and specificity. In addition, as the content of inhibiting factors and contaminants can be highly variable between specimen types, specialised optimisation of conditions, such as concentration of the chemical or enzyme used, heat treatment temperature, and time, is required for each different intending specimens. While these methods may offer alternatives to avoid RNA extraction and streamline SARS-CoV-2 NAATs, it is important to consider that these methods have been explored and optimised only under research conditions with readily available equipment such as heat blocks and real-time PCR systems. Consequently, in the foreseeable future, extraction-free SARS-CoV-2 NAATs may still require dedicated laboratory space until the development of novel and more deployable platforms which can incorporate alternative extraction-free sample preparation and NAAT detection. Therefore, it is advisable to test and validate these workflows following the applicable national regulatory procedures before using them clinically together with approved NAAT kits for the surveillance or diagnosis of SARS-COV-2 infections. Similarly, biosafety precautions must be taken into consideration before implementing any of these strategies, as many do not inactivate infectious material. Guidelines such as the CDC lab and point-of-care biosafety guidelines for handling and processing COVID-19 samples are recommended to minimize the risk of infection [42,43].

Though all the methods listed here have been reported to streamline NAATs by omitting RNA extraction steps, the superiority of one over the other has not been tested simultaneously and remains unknown. Reported sensitivities and specificities by these studies, as well as details on their methodologies, are reported in Supplementary Table S1 but have not been considered due to a series of potentially confounding variables that may bias their comparison. These include differences in experimental design, specimen used, NAAT kits, and instruments used.

The impact of these methods on other NAATs that make use of similar respiratory specimens offers great opportunities for the development of cheaper diagnostic and point-of-care tests, but further investigation is required. In the event of future pandemics caused by respiratory pathogens, extraction-free NAATs can help mitigate the immediate impact of in vitro diagnostic kit shortages on testing capacity and public health measures. In contrast, the impact of these strategies on other type of specimens such as stool, urine, and blood products cannot be extrapolated as these types of specimens may contain other types and amounts of inhibitors, proteolytic agents, and interfering substances.

## 4. Conclusions

COVID-19 management has been impacted by shortages in in vitro diagnostic kits and supplies during the course of the pandemic, predominantly in underprivileged regions. The first diagnostic test developed and deployed for the identification of COVID-19 cases was RT-PCR. SARS-CoV-2 testing by RT-PCR is often preceded by expensive and laborious RNA extraction workflows. Sample preparation alternatives such as an increase in sample input, dilution of the sample, precipitation of nucleic acids, heating of the sample, or addition of chemical and biological agents with proteolytic, mucolytic, and inhibitory properties prior to amplification have been found useful. These methods are successful at bypassing the typical RNA extraction, provide acceptable results, and can advance the development of point-of-care NAATs. Further optimisation and validation of these methods can facilitate not only ramping up the molecular diagnosis of SARS-CoV-2 in underprivileged and privileged regions but can also serve as alternatives to overcome shortages in future pandemics.

## Figures and Tables

**Table 1:** Strategies shown to facilitate the use of direct respiratory samples in NAAT of SARS-CoV-2.

Sample Type	NAAT/Detection Method	Sample Preparation Strategy	Ease of Implementation	Reference
Nasopharyngeal and nasal swab in UTM	RT-PCR	Increase in sample input combined with the use of enzymes with high tolerability to inhibitors *	Very easy to implement as no sample treatment is required.	[7]
Nasal and throat swabs suspended in nuclease-free water	RT-PCR, RT-LAMP	Increased input volume of swabs eluted in nuclease-free water or saline *	Very easy to implement as no sample treatment is required.	[8,39]
NP swabs in UTM, PBS, Hanks medium, DNA/RNA shield	RT-PCR	Precipitation of sample with PEG/NaCl combined with heat treatment at 70 °C for 30 min.	Laborious methodology as precipitation involves more than one step. A heating source is required for this method.	[10]
Heat-inactivated nasopharyngeal swab-UTM eluates	RT-PCR	Precipitation of template with 1.1 volumes of isopropanol, incubation at −20 °C for 30 min and centrifugation, ethanol addition, and centrifugation.	This method involves several steps, including centrifugation and a freezer.	[11]
Swab in viral transport medium	RT-PCR	Reduced input volume of swabs eluted in viral transport medium *	Very easy to implement as no sample treatment is required.	[14]
Nasopharyngeal swab in UTM	Fluorescence RT-LAMP, RT-PCR	Dilution of sample in RNase-free water *	Very easy to implement as no sample treatment is required.	[16,17]
Nasopharyngeal swabs and saliva	RT-PCR and RT-ddPCR	Elution of swabs into Chelex-TED buffer (50% Chelex-100, TE buffer, DMSO) or addition to saliva, followed by heat treatment at 98 °C for 5 min and centrifugation.	Difficult to implement as this method involves several steps, and relies on a centrifuge and a heating source.	[18]
Sputum and nasal exudate	Portable RT-PCR	Treatment of sample with sputasol and the RNAse inhibitor RNAseOUT™ *	Easy to implement as the sample can be treated in one step.	[15]
Saliva	Colorimetric RT-LAMP	Combination of proteinase K treatment, heat inactivation, and RNAsecure treatment.	Challenging to implement as several sample preparation methods are involved and Proteinase K treatment requires a final step to denature the enzyme.	[21]
Saliva and swabs	Colorimetric RT-LAMP	Addition of carrier nucleic acid, treatment with RNase inhibitors, and increase in the reaction volume *	Easy to implement as treatment of the sample can be done in one step.	[22]
Saliva and Nasopharyngeal swabs	RT-PCR and RT-LAMP	Elution of swab or mixing of saliva with RNA stabilization buffer (TCEP, EDTA, Chelex, and RNasecure in Tris buffer) followed by 95 °C 15 min heat inactivation and cooling.	Although several sample preparation methods are involved, this strategy can be done in two steps.	[19]
Saliva	RT-LAMP	1:1 dilution in Mucolyse (DTT), followed by dilution in 10% (*w*/*v*) chelex 100 resin and 98 °C heat treatment for 2 min.	Several sample preparation methods and steps are involved making it challenging to implement.	[20]
Saliva or Nasopharyngeal swab eluted in saline or PBS	Colorimetric RT-LAMP	Combination of treatment with a reducing agent (TCEP/EDTA) and heat treatment at 95 °C for 5 min.	Relatively easy to implement as treatment of the sample involves a two-step process. However, a heating source is required.	[23]
Saliva, nasopharyngeal and oropharyngeal swabs eluted in saline or UTM	RT-PCR	Proteinase K followed by heat inactivation at 95–98 °C for 5 min.	Moderately easy to implement; however, denaturing Proteinase K at high temperature is essential.	[24,25,26,27,28]
Nasopharyngeal, oropharyngeal swab in transport medium, saline, PBS, or water. Saliva	RT-PCR and RT-LAMP	Several heating conditions from 65 °C to 98 °C for periods of 5 to 30 min.	Relatively easy to implement; however, this strategy requires a heating source and optimization of the heating conditions.	[10,13,23,29,30,31,32,33,34,37,40,41]
Nasopharyngeal swabs	RT-PCR	Thermal shock of the sample at 95 °C for 5 min followed by 4 °C for 10 min.	Relatively easy to implement; however, this strategy requires both a heating source and an active cooling source.	[36]
Nasopharyngeal swabs in universal transport media	RT-PCR	Combination of heat treatment (65 °C for 10 min) and increase in sample input volume.	Relatively easy to implement; however, this strategy requires a heating source.	[30]
Saliva	RT-PCR	Lysis in TBE buffer and tween-20 combined with heat treatment at 95 °C for 30 min.	Relatively easy to implement; however, this strategy requires a heating source.	[32]
Nasopharyngeal swabs and gargle lavage	Fluorescence and Colorimetric RT-LAMP	Combination of Quickextract and heat treatment at 95 °C for 5 min, supplemented with carboxylated magnetic beads to enrich target RNA.	Several sample preparation methods and steps are involved making it challenging to implement.	[41]

* This sample preparation strategy is unlikely to inactivate infectious material and may pose a risk to the operator if appropriate biosafety measures are not in place.

## Data Availability

Not applicable.

## Supplementary Materials

The following supporting information can be downloaded at: https://www.mdpi.com/article/10.3390/v14061311/s1, Table S1: Strategies shown to facilitate the use of direct respiratory samples in NAAT of SARS-CoV-2 (extended table).
