# Supplementary material for: Strategies That Facilitate Extraction-Free SARS-CoV-2 Nucleic Acid Amplification Tests
Source: Viruses. 2022 Jun 15;14(6):1311. doi: 10.3390/v14061311 (PMC9230587; doi:10.3390/v14061311)
Supplement: Supplementary file 1 [file viruses-14-01311-s001.zip › viruses-1761338-supplementary.pdf]

viruses

MDPI

Supplementary Materials:

Supplementary Table S1. Strategies shown to facilitate the use of direct respiratory samples in NAAT of SARS-CoV-2 (extended table)

Sample type	NAAT / detection method	Sample preparation strategies	Analytical or clinical sensitivity	Specificity	Reference
Nasopharyngeal and nasal swab in UTM	RT-PCR	14 ul of sample input, used enzymes with high tolerability to inhibitors.<br>14 ul input into a 30 ul reaction	550 viral RNA copies/ml	Not reported	[7]
Nasal and throat swabs suspended in nuclease free water	RT-PCR	Elution in nuclease free water and increased input volume of 10ul into 25 uL reaction.	93% overall if tested in duplicates, 100% for high to moderate viral nucleic acid load	Not assessed	[8]
Nasopharyngeal, oropharyngeal swabs	RT-LAMP	Dry swab eluted in 0.5 mL saline and shaken vigorously for 30 minutes.<br>Higher input volume (11.5 ul input into 25 ul reaction)	95.6%	99.4%	[40]
Nasopharyngeal swabs in UTM, PBS, Hanks medium, DNA/RNA shield	RT-PCR	Precipitation with PEG/NaCl and Heat treatment at 70 ^{o}C for 30 minutes.	100% relative to gold standard method	100% relative to gold standard method	[10]
Heat inactivated nasopharyngeal swab-UTM eluates	RT-PCR	Mixing with 3M sodium acetate, 5mg/ml linear acrylamide, followed by precipitation with 1.1 volumes of isopropanol, incubation at -20C for 30 minutes and centrifugation, ethanol addition and centrifugation.	Not reported	Not reported	[11]
Swab in viral transport medium	RT-PCR	Low input volume (2ul) into 20 uL reaction	98% relative to std method	100%	[15]

Nasopharyngeal swab in UTM	Fluorescence RT-LAMP	1 in 5 dilution of sample in water	87% sensitivity with limit of detection of 54 $TCID_{50}$/ml	100%	[17]
Nasopharyngeal swabs in UTM	RT-PCR	1 in 4 dilution of specimen in sterile RNase free water prior to the addition of the template to the mastermix	74.0% and 82.1% with the Allplex TM SARS-CoV-2 assay (tested with 139 NPS samples) and the Allplex TM SARS-CoV-2/FluA/FluB/RSV assay (tested with 69 NPS samples)	100%	[18]
Nasopharyngeal swabs and saliva	RT-PCR and RT-ddPCR	Elution of swabs into Chelex-TED buffer (50% Chelex-100, TE buffer, DMSO) or addition to saliva, followed by heat treatment at 98C for 5 minutes and centrifugation to collect supernatant.	1 copy/ul	Not reported	[19]
Sputum and nasal exudate	Portable RT-PCR	1:1 dilution with sputasol and treatment with the RNAse inhibitor RNAseOUT™. Reduced template input volume to 2ul in a 20 ul reaction.	6 RNA copies per reaction	Not assessed	[16]
Saliva	30 minutes Colorimetric RT-LAMP	Saliva is heat treated at 65C for 15 minutes followed by 95C for 15 minutes. Sample is then treated with Proteinase K and RNAsecure.	85% sensitivity	100%	[22]
Saliva and swabs	Colorimetric RT-LAMP	Addition of carrier nucleic acid, RNase inhibitors, and increasing the reaction volume	~2 viral copies / ul of saliva. 97% sensitivity	100%	[23]
Saliva and Nasopharyngeal swabs	RT-PCR and RT-LAMP	Elution of swab or mixing of saliva with RNA stabilization buffer (TCEP, EDTA, Chelex, and RNasecure in Tris buffer) followed by 95C 15 minutes heat inactivation and cooling.	95%	100%	[20]

Saliva	RT-LAMP	1:1 dilution in Mucolyse (DTT), followed by dilution in 10% (w/v) chelex 100 resin and 98C heat treatment for 2 minutes	1 X10^1 – 1 X 10^2 copies/ ul		[21]
Nasopharyngeal and oropharyngeal swabs eluted in saline	RT-PCR	Proteinase K followed by 98C for 5 minutes	91% accuracy with standard procedure. Average increase of 5.64 Ct	Not assessed	[25]
Saliva	RT-PCR	Addition of 60 ug Proteinase K followed by incubation at 37C for 5 minutes and 95C heat treatment for 5 minutes.	9441 copies/mL 97% sensitivity in first days of symptoms and 82% sensitivity in asymptomatic cases	97-98%	[26]
Nasopharyngeal swabs	RT-PCR	Addition of Proteinase K followed by incubation at 55C for 15 minutes and heat treatment at 98C for 5 minutes	10 RNA copies 90%	100%	[27]
Nasopharyngeal swabs	RT-PCR	Addition of 10 ul of 10 mg/ml Proteinase K followed by incubation at 55C for 15 minutes and heat treatment at 98C for 5 minutes	99%	99%	[28]
Nasopharyngeal swabs	RT-PCR	Proteinase K treatment (3 μg/μL) followed by incubation at 56˚C for 10 minutes and heat denaturation at 98˚C for 5 minutes.	100%	100%	[29]
Pharyngeal swab specimens in Amies medium	Colorimetric RT-LAMP (30 min run-time)	Heating at 95°C for 5 minutes.	86% sensitivity	99.5%	[30]
Saliva	Colorimetric RT-LAMP	Heat treatment 95C 10 minutes.	100 virions/ul	97% positive agreement and 100% negative agreement	[41]

Nasopharyngeal swabs in transport medium	RT-PCR	Heat treatment 95°C for 5 minutes.<br>1-4 ul of sample input into a final reaction volume of 20 ul.	~96 % sensitivity depending on primers used and relative to Roche Cobas 6800 analyzer	~100 %	[14]
Saliva or Nasopharyngeal swab eluted in saline or PBS	30 min Colorimetric RT-LAMP	Addition of TCEP/EDTA (2.5mM/1mM) with addition of NaOH to adjust pH) and Heat treatment 95°C for 5 minutes.<br>RT-LAMP tolerates at least 5 ul of input material into a 25 ul reaction	50 viral copies/ul of sample achieved 85% sensitivity relative to RT-PCR	Not reported	[24]
Nasopharyngeal swabs in UTM or dry swabs eluted in PBS	RT-PCR	Heat treatment at 95°C for 10 minutes and 1:10 sample dilution.<br>In-house RT-PCR protocol	100% sensitivity relative to gold standard method	100%	[32]
Saliva	RT-PCR	Combination of saliva with collection TBE buffer. Heat 95C 30 minutes + TBE buffer and Tween-20	500-1000 viral particles/ml, comparable to std method	98.9%	[33]
Oro- and nasopharyngeal samples in UTM or molecular water	RT-PCR	Heat inactivation 95C for 5 minutes	100% accurate relative to std method when targeting the N gene	Not assessed	[34]
Nasopharyngeal swabs eluted in M6 viral transport media	RT-PCR	Heat treatment 95C for 10 minutes and lower input (3ul vs 5ul) volume	92% accuracy when compared to std method	Not assessed	[38]
Nasopharyngeal swabs in universal transport media	RT-PCR	Nasopharyngeal swabs in UTM and diluted 1:4 in nuclease free water<br>65C for 10 minutes<br>8ul input into 20 ul reaction	Analytical sensitivity corresponding to 6.6x10^3 RNA copies/ml<br>95% sensitivity and 98.5% accuracy relative to std method	99%	[31]
Oropharyngeal swabs eluted in	RT-PCR	Heat treatment 98C for 5 minutes<br>5ul input into 25 ul reaction	97.4% sensitivity, 98.3% accuracy	100%	[35]

saline or transport solution					
Nasopharyngeal swabs and gargle lavage	Fluorescence and Colorimetric RT-LAMP	Swab eluates or gargle lavages (10 ml HBSS or saline) mixed with 2X Quickextract.<br>Heat treatment at 95C for 5 minutes. dUTP and thermolabile Uracil DNA Glycosylase used to prevent carry-over contamination.<br>Carboxylated magnetic beads to enrich RNA and increase sensitivity.<br>2ul input into 20 ul reaction	~50 copies per reaction (crude sample)<br>~5 copies per reaction (bead enrichment)	100%	[42]
Nasopharyngeal swabs	RT-PCR	Thermal shock of sample at 95C for 5 minutes followed by 4C for 10 minutes.	Not reported	Not reported	[37]

NAAT, nucleic acid amplification test; RT-LAMP, reverse transcription loop-mediated isothermal amplification; RT-PCR, reverse transcription polymerase chain reaction; RT-ddPCR, reverse transcription digital droplet polymerase chain reaction; Ct, cycle threshold; TCEP/EDTA, tris(2-carboxyethyl)phosphine/Ethylenediaminetetraacetic acid; DTT, dithiothreitol; UTM, universal transport media; PBS, phosphate buffered saline; $TCID_{50}$/ml, Median Tissue Culture Infectious Dose/ml; TBE buffer, Tris-Borate-EDTA buffer; HBSS, Hanks' Balanced Salt Solution; dUTP, deoxyuridine triphosphate.
